# High-Resolution Remote Sensing Image Classification with RmRMR-Enhanced Bag of Visual Words

**DOI:** 10.1155/2021/7589481

**Published:** 2021-04-15

**Authors:** Suting Chen, Liangchen Zhang, Rui Feng, Chuang Zhang

**Affiliations:** ^1^CICAEET, School of Nanjing University of Information Science & Technology, Nanjing 210044, China; ^2^Jiangsu Key Laboratory of Meteorological Observation and Information Processing, Nanjing University of Information Science & Technology, Nanjing 210044, China

## Abstract

A ReliefF improved mRMR (RmRMR) criterion-based bag of visual words (BoVW) algorithm is proposed to filter the visual words that are generated with high information redundancy for remote sensing image classification. First, the contribution degree of each word to the classification is represented by its weighting parameter, which is assigned using the ReliefF algorithm. Next, the relevance and redundancy of each word are calculated according to the mRMR criterion with the addition of a dictionary balance coefficient. Finally, a novel dictionary discriminant function is established, and the globally discriminative small-scale dictionary subsets are filtered and obtained. Experimental results show that the proposed algorithm effectively reduces the amount of redundant information in the dictionary and better balances the relevance and redundancy of words to improve the feature descriptive power of dictionary subsets and markedly increase the classification precision on a high-resolution remote sensing image.

## 1. Introduction

Remote sensing classification is the process of discriminating ground objects according to the spatial features, spectral features, and temporal features in a remote sensing image to assign an identifier to each element in the image. Recently, the advancement in high-resolution remote sensing technology has delivered an image that contains rich ground object information and complex spatial relations, which feature high dimensionality, high resolution, and a large volume of data [1]. Therefore, a current research focus is determining how to efficiently extract and classify the desired information from the massive amount of data in a remote sensing image.

The traditional statistical pattern-based classification algorithm considers independent pixels and thus cannot utilize the spatial structural features such as texture, scale-invariance, and shape of a high-resolution remote sensing image and does not comply with the distribution law of the target space, resulting in multiple discrete isolated points, i.e., the “salt and pepper phenomenon” [2]. This makes subsequent classification difficult and cannot achieve a satisfactory classification precision. In view of these problems, the object-oriented high-resolution remote sensing image classification methods have attracted extensive attention and research interest. In such methods, BoVW is a representative method that is based on the concept of clustering the low-level features into visual words and associating these visual words with the image semantics through their distribution to represent the image content [3]. This algorithm efficiently solves the single-feature description and the “semantic gap” between high- and low-level features. However, since the visual words extracted from the classic BoVW algorithm are subject to redundancy, the visual dictionary must be optimized to yield the best image classification result. Yang et al. [4] joined the construction of the visual dictionary with classifier training and synchronously updated the words and the parameters of the classifier. The advantage of this approach is that the learned visual dictionary will be more suitable for the classifier, but the generalization ability is weak and shows poor classification performance. Epshtein and Ullman [5] deleted the words that contain less mutual information with classes by calculating the mutual information between words and the image; Kim et al. [6] proposed an entropy-based visual word filtering algorithm to retain those words with lower entropy between classes. Such methods only consider the relevance between words and classes and ignore the redundancy between words. Thus, the dictionary that is based on the maximum relevance between words and classes may not always be the best dictionary, but the maximum relevance between the whole dictionary and classes should be considered.

Given the problems described above, a ReliefF improved mRMR (RmRMR) criterion-based BoVW algorithm is proposed here according to mRMR criterion [7] and ReliefF algorithm [8]. In addition, this paper filters the words with less redundancy between words and large relevance between words and classes, constructs the globally discriminative small-scale dictionary, exploits the image-rich data resource, and reduces the computational complexity to achieve high-resolution remote sensing image classification in a fast and accurate manner. To verify the effectiveness of the proposed algorithms, the evaluation experiments are carried out on a high-resolution remote sensing image.

## 2. RmRMR-Enhanced Bag of Visual Words

### 2.1. Overall Framework

**Figure 1:**
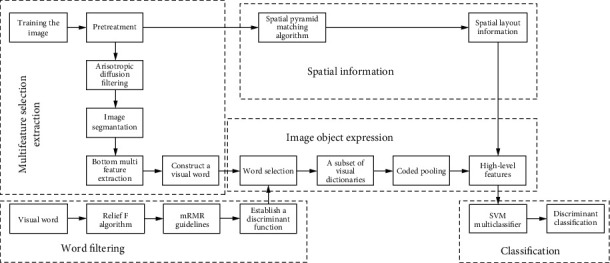
Process flow of the proposed algorithm.

As shown in Figure 1, the image is preprocessed, the edge details were enhanced, and the noise is eliminated by anisotropic diffusion filtering. Then, the image is segmented using the watershed-based image segmentation algorithm to obtain the objects on an image. The texture, shape, KAZE, and other multifeatures of the object are extracted. We next use K-means clustering to integrate the features of a specific object. In the next, the visual words are constituted, and the feature weights of the words are calculated using ReliefF for word selection. In the following, the globally discriminative small-scale dictionary subset is extracted according to the weighted mRMR criterion. As the last step, the obtained high-level image semantic eigenvector is used as an input for the SVM multiclassifier to complete the classification training.

### 2.2. RmRMR-Based BoVW Model

**Figure 2:**
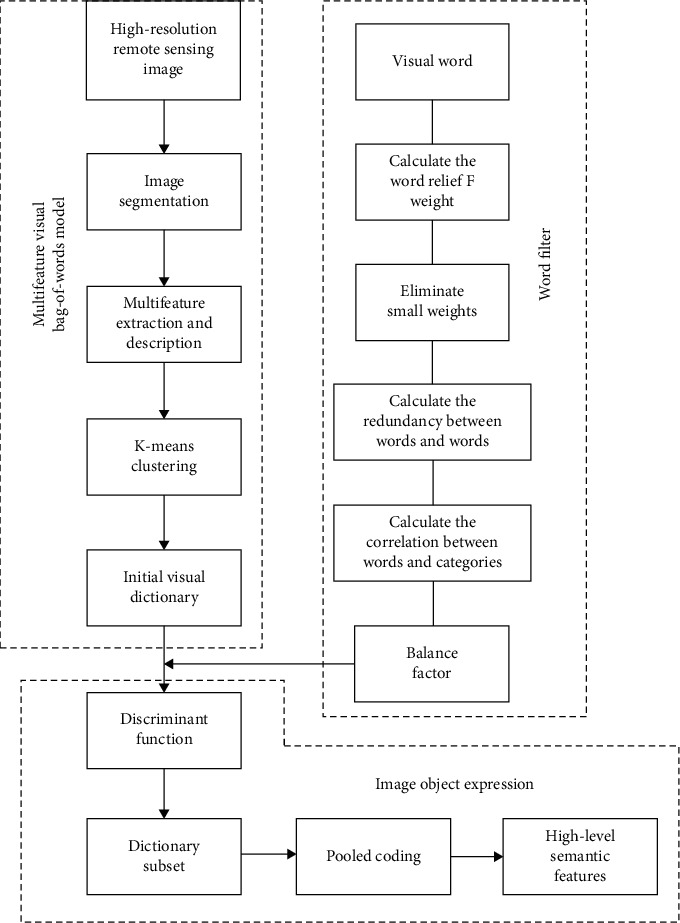
Process flow of RmRMR-based BoVW algorithm.

This work introduces the BoVW model to fuses the texture, shape, and KAZE into the low-level features and replaces the conventional method of single-feature representation for feature extraction. In addition, we apply the mRMR criterion (maximum relevance between word and class with minimum redundancy between words) to remote sensing image classification and globally considers the relevance and redundancy in the visual dictionary, Additionally, the proposed method adds the weight parameters of visual words using ReliefF and proposes a new dictionary discriminative power evaluation function to find a dictionary subset with strong discriminative power and less redundant information. The visual dictionary is optimized in this manner, and the process flow of the algorithm is shown in Figure 2.

The difference between this paper and the previously published research and proposed model of Deng et al. titled “Multi-Level Image Representation for Large-Scale Image-Based Instance Retrieval” is adding the step “Calculate the redundancy between words and words.”

### 2.3. Multifeature BoVW Model

To eliminate the gap between low- and high-level semantic features, the middle-level features will be used to count and cluster the low-level features to establish a link with the high-level semantic features [9]. Bag of visual words (BoVW) is a type of middle-level feature representation that has been extensively used as an alternative general and accurate image feature representation for image analysis and processing. In this paper, BoVW is introduced to represent the features of high-resolution remote sensing images to bridge the gap between low- and high-level features. The BoVW-based image object expression can be divided into the following steps:

 
*Step 1*. Spatially segmenting the image *I* needs to be processed, and assume that the image is segmented into *M* levels, and the *m*th level is segmented into 4^*m*−1^ subregions
 
*Step 2*. Extracting the texture, shape, and KAZE from the subregions in the subspace: In this work, multifeature fusion is performed by a serial method, and the fused low-level image features are used as the objects for constructing visual words
 
*Step 3*. Building a visual dictionary: The extracted low-level features are used as the set to be clustered *X*={*x*_1_, *x*_2_,…, *x*_*n*_} using the K-means clustering algorithm. It selects *k* points as the clustering centers, which is set to *μ*_1_, *μ*_2_, *μ*_*k*_,…, ∈*R*^*n*^, and calculates the Euclidean distances between every eigenvector and the clustering centers. It classifies the nearest distances into a group according to the following equation:

(1) $$\begin{array}{c} c^{\left(i\right)}=\arg\;\min{\left\|x^{\left(j\right)}-\mu_{j}\right\|}^{2}. \end{array}$$

As each class is a visual word, a visual dictionary of size *k* is constructed. The clustering parameter *k* will impact the performance of the visual dictionary, which should be acquired through experimental comparison according to the classified image features.

A single feature will cause the problem that the image content is not comprehensive and accurate. The multifeature method integrates image texture features, shape features, KAZE features, etc. as the underlying features, replacing the traditional single-feature description method, and making up for the single-feature description method in the independent expression of high-resolution remote sensing images, which cannot fully express image information.

### 2.4. Word Selection Method of the RmRMR

In the BoVW, the visual dictionary is obtained by learning the distribution of the training image. Significantly, the quality of this dictionary plays an important role in the image classification results. For example, excessive content in the dictionary or excessive similar words will add computational complexity and data redundancy, while sparse content in the dictionary will misclassify different targets into a group, and thus, it reduces the image classification precision. Specifically, optimizing the visual dictionary of the BoVW can achieve a large margin for image classification results. In general, an excellent visual dictionary should have two characteristics: (1) each visual word is highly relevant to an image class; (2) the redundancy between words is minimal; i.e., the presence of fewer redundant words results in lower computational complexity of the algorithm. The screening of visual words is an efficient way to optimize the visual dictionary, which is intended to select a few globally discriminative words from the initial dictionary to constitute a dictionary subset.

The mRMR algorithm does not adequately describe the content of the image for a visual dictionary that is too small, resulting in different image objects being classified into one category, which reduces the classification accuracy. The use of the unimproved algorithm is that the redundant features always exist and are not deleted, and redundant features may be mapped to other words, which may lead to errors in the mapping between words and features. Visual words cannot accurately describe image information, resulting in unsatisfactory classification results.

The mRMR visual residual selection algorithm improved by the K-means algorithm can weight words, retain the words with high weights that contribute to the classification, and at the same time fully consider the redundancy between words and the correlation between words and categories, thereby, improving the accuracy of image classification. Using the K-means algorithm can correctly classify the different features of the image and has good results.

In this thesis, the visual dictionary is optimized through word filtering, and the subset of the visual dictionary with the greatest contribution to the classification performance is selected. ReliefF is a feature weighting algorithm that will give a high weight to a word with a large contribution to the classification, but this method cannot eliminate the redundancy between words. The mRMR criterion can screen out the subset of the dictionary that has maximum relevance between word and class and the minimum redundancy between words; however, the weight coefficients of single words will not be obtained, nor will the effects of different words in the dictionary subset on the classification be reflected. Given the advantages and disadvantages of the two algorithms described above, we implement word filtering by RmRMR, and the specific steps are as follows:

*Step 1*. Visual word weighting: The algorithm defines *V*=[*v*_1_,…,*v*_*k*_]^*T*^ as the initial visual dictionary and *V* as a matrix of size *K* × *D*, where *K* denotes the number of words in the dictionary and *D* is the dimensionality of the image features. The relevance weight *w* between each word and image class in the initial visual dictionary is calculated using ReliefF. The equation is described as follows:
  (2) $$\begin{array}{c} w\left(a\right)\;⟸\;w\left(a\right)-\overset{k}{\underset{j=1}{\sum }}\frac{\text{diff}\left(a,v_{i},v_{j}\right)}{m-k}\\ +\underset{c\neq \text{class}\left(v_{i}\right)}{\sum }\frac{\left\{\left(P\left(c\right)|/|\left(1-P\left[class\left(v_{i}\right)\right]\right)\right)\times \sum_{j=1}^{k}\left\{\text{diff}\left[a,v_{i},m_{j}\left(c\right)\right]\right\}\right\}}{\left(m-k\right)}, \end{array}$$
where *m* denotes the number of iterations, *k* is the number of nearest samples and denotes the distance of the randomly sampled words *v*_*i*_ and *v*_*i*_ relative to the word *a*, and *P*(*c*) is the probability of the *c*th class of targets; the word *a* = 1,…, *d* is cyclic, set *w*(*a*) = 0.
Small weight word deletion: A dictionary subset *V*_*n*_ with a weight matrix *w*_*n*_ is obtained by calculating the relevance weight *w* between each word and image class and removing the weight of the smallest features. The dictionary subset is weighted to obtain *V*_*m*_=*V*_*n*_ · *w*_*n*_.
*Step 2.* Correlation and redundancy calculation: The equation for the relevance between the word *v*_1_ and the class *c* is
  (3) $$\begin{array}{c} Rlv\left(v_{1},c\right)=I\left(v,c\right). \end{array}$$
According to the above equation, the relevance between the dictionary *V* and the image class *c* is
  (4) $$\begin{array}{c} Rlv\left(V,c\right)=\frac{1}{\left|V\right|}\sum_{v_{j}\in V}I\left(v,c\right), \end{array}$$
where *I* is the mutual information function:
  (5) $$\begin{array}{c} I\left(v_{i},c\right)=\underset{c\in \left(0,1\right)}{\sum }p\left(v_{i},c\right)\log\frac{p\left(v_{i},c\right)}{p\left(v_{i}\right)p\left(c\right)}\\ +\underset{c\in \left(0,1\right)}{\sum }p\left({\bar{v}}_{i},c\right)\log\frac{p\left({\bar{v}}_{i},c\right)}{p\left(v_{i}\right)p\left(c\right)}. \end{array}$$
Mutual information [10] as a criterion for the relevance between word and class will allow the algorithm to select the discriminative words in a more accurate manner. When the visual words randomly or uniformly appear in the images of different classes, the value of the mutual information will be close to 0; when the frequency differences among visual words that appear in the images of different classes are sharp, the value of the mutual information will be higher.
The mean value of the relevance between all words and classes in the dictionary can be calculated by equation 4. The top-*N* words with maximum relevance are selected to ensure that the mean value of the visual dictionary subset that they comprise will be optimal. However, since redundancy definitely exists between the words in such a set, the dimensionality reduction here is necessary. Then, the redundancy of the words is incorporated into the dictionary discriminant function. The redundancy between words, *v*_*i*_, is calculated as follows:
  (6) $$\begin{array}{c} R\;dd\left(v_{j}\right)=\frac{1}{\left|V\right|-1}\sum_{v_{j}\in V,v_{i}\neq v_{j}}I\left(v_{i},v_{j}\right). \end{array}$$
From the above equation, the redundancy between words in the visual dictionary is
  (7) $$\begin{array}{c} R\;dd\left(V\right)=\frac{1}{{\left|V\right|}^{2}}\sum_{v_{i},v_{j}\in V}I\left(v_{i},v_{j}\right). \end{array}$$
*Step 3*. Balance coefficient of fusion: Set the threshold *d*, i.e., the size of the dictionary. The word that is the most relevant to the class is determined by equation (4) and added to the dictionary subset *V*, and then the next word is selected by the mRMR criterion until *d* words have been selected. The discriminant function is as follows:
  (8) $$\begin{array}{c} \max_{v_{i}\in V-V_{m}}\left[I\left(v_{i},c\right)-\frac{1}{m}\sum_{v_{j}\in V_{m}}I\left(v_{i},v_{j}\right)\right]. \end{array}$$

The words in the initial visual dictionary are weighted by the above equation. Specifically, the words with the greatest contributions to the classification are selected, and the visual dictionary subset with the maximum relevance between word and class with the minimum redundancy is extracted using the mRMR algorithm. To balance the relevance and redundancy in the dictionary and obtain a better dictionary subset, a weight coefficient *α*(0 ≤ *α* ≤ 1) is introduced, and the above equation is transformed as follows:

(9) $$\begin{array}{c} \max_{v_{i}\in V-V_{m}}\left[\alpha I\left(v_{i},c\right)-\left(1-\alpha \right)\frac{1}{m}\sum_{v_{j}\in V_{m}}I\left(v_{i},v_{j}\right)\right]. \end{array}$$

The larger the value of *α*, the more concerned the algorithm is with the relevance between word and image class; when *α* is smaller, the algorithm limits the relevance between words and minimizes it as much as possible. The choice of *α* is related to the image class and requires optimization through experimental comparison or empirical selection.

## 3. Realization of the Classification Model

In the proposed model, the purpose of image block segmentation is to acquire the image regions of interest, and the common methods include random sampling, uniform gridding, and saliency detection. In the BoVW, the extraction of low-level object features is the first step of the algorithm, which will significantly impact the algorithm's performance. In this sense, it is essential to select a feature descriptor with robustness, general representation of features, and high accuracy; optional features include texture, shape, and KAZE [11]. At the stage of quantitative clustering, the K-means clustering method is usually used to generate the visual dictionary, but its size depends on the clustering parameters (the number of clustering centers), as different values may have significantly different impacts on the subsequent image classification. Therefore, it is necessary to select the optimal parameter value through experiments. The last stage of the BoVW approach is to calculate the similarity between objects and visual words, and generally, the Euclidean distance is used, and each object is mapped to a word to obtain all words in the histogram representation.

After obtaining a subset of the visual dictionary, the expression of the image object needs to be realized. According to the pooling function *z* = *F*(*s*), the high-dimensional eigenvectors generated after clustering will be aggregated into an independent vector, and the lengths of different eigenvectors will be made uniform for classification using linear SVM to enhance the antinoise properties and the robustness of the algorithm. The average pooling function is selected:

(10) $$\begin{array}{c} z_{j}=\frac{1}{M}\overset{M}{\underset{i=1}{\sum }}\left|s_{ij}\right|, \end{array}$$

where *M* is the number of eigenvectors and *S* is the encoded matrix.

Here, an image is represented by constructing an unordered set of visual vocabularies through K-means clustering, but the spatial layout information in the low-level features is lost, which may lead to misclassification of the same object in different orders. Therefore, Spatial Pyramid Matching (SPM), which was proposed by Lazebnik et al. [12], is introduced to add the image spatial layout information so that the problem of missing spatial information in the BoVW is effectively solved [13].

SPM spatially segments an image into a pyramid form. In this paper, *L* = 2 is selected as the construction level for the image's spatial pyramid and *k*_*l*_=(1/2^*L*−*l*+1^) as the weight value of level *l*. If *L* = 0, the image is not segmented and the global feature histogram of the image is extracted with a weight value of 1/4; if *L* = 1, the image is segmented into four blocks, and the feature histogram for each subblock is extracted with a weight value for each subregion of 1/2; if *L* = 2, the image is segmented into 16 blocks, and the feature histogram of each subblock is extracted with a weight value of 1/4. Then, the spatial feature histograms from three levels are weighted and combined into the image's spatial pyramid representation. The SPM-based BoVW equation is described as follows:

(11) $$\begin{array}{c} K^{M}=\sum_{i=1}^{V}\sum_{m=1}^{M}\alpha_{m}\sum_{d=1}^{4^{m-1}}h_{i}^{m}\left(d\right), \end{array}$$

where ∑_*d*=1_^4^*m*−1^^*h*_*i*_^*m*^(*d*)is the histogram representation of the *i*th word in each subregion at the *m*th level, *d* denotes the subregion number^,^*α*_*m*_ is the weight of each image level, and *V* denotes the number of visual words, i.e., the number of clustering centers *k*.

In the last step, the obtained spatial layout information and the high-level features encoded from image features are fused as the final input of the classifier through the following linear classification function [14]:

(12) $$\begin{array}{c} f\left(z\right)={\left(\sum_{i=1}^{n}\alpha_{i}z_{i}\right)}^{T}z+b=\omega^{T}z+b. \end{array}$$

## 4. Experiments

### 4.1. Implementation Details

In order to validate the effectiveness of the proposed algorithm, the US GeoEye-1 remote sensing image is selected as the source of the experimental dataset, which has a spatial resolution of 0.4 m. The GeoEye-1 high-resolution satellite has advantages of large-scale precision mapping and fast detection as well as parsing of ground objects, and it provides high-quality services to users around the globe. The selected experimental image is the size of 2500 × 2100 pixels with geometric correction and contains five major types of ground objects: water, farmland, building, road, and bare land. In addition, 150 patches are randomly sampled from each type of object in the remote sensing experimental image to train the classification algorithm and evaluate the classification performance. The experimental image is shown in the figure hereinafter. Our model is implemented with PyTorch v1.0 and trained on a workstation with one NVIDIA Titan X GPU of 12 GB memory, CUDA 10.0, and cuDNN 7.4.1 (https://pytorch.org/).

### 4.2. Hyperparameters Optimization

In order to obtain the key adjustable parameters that appear in the proposed algorithm, including the scale of the visual dictionary, the number of training samples, and the weighting factor of the RmRMR criterion, we empirically select different hyperparameters of our model configuration during training, and these optimal hyperparameters in the experiment are used for inference through comparison.

The scale of the visual dictionary is determined by the clustering parameter *k*, which will affect the classification capacity of the proposed algorithm. Furthermore, to validate the effectiveness of the RmRMR criterion that is proposed here for the selection of visual words, this section compares the RmRMR algorithm and the K-means clustering algorithm in terms of the classification accuracy and examines the effectiveness of different visual dictionary scales on the classification for these two algorithms. The clustering hyperparameter in the K-means algorithm *k* and the size of dictionary subset in the RmRMR algorithm *d* are assigned values ranging from 200 to 1,200, while other hyperparameters are kept the same, and the average classification accuracy for 20 experiments is used as the evaluation result.

**Figure 3:**
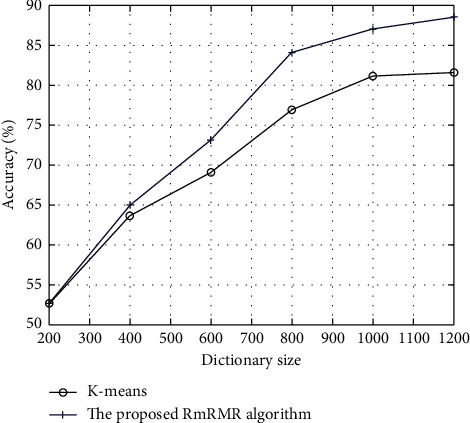
Comparison of experiment results for different dictionary scales.

From Figure 3, when the scale of the visual dictionary is small, the average classification accuracies of the two algorithms are relatively low. This is because the image content is inadequately described if the visual dictionary is not complete, so that different objects in the image may be easily classified in a group, thus reducing the classification precision. As the scale of the visual dictionary increases, the classification accuracy of the algorithm is sharply increased, and the growth rate in the RmRMR algorithm is higher. This suggests that the proposed algorithm has a distinctive advantage when the number of words in the visual dictionary is greater than approximately 600. This is because the size of the dictionary is controlled with the K-means algorithm, so the redundant features are always included in the dictionary, which may be mapped to other words. Furthermore, the mapping between words and features may be deviant, and the visual words cannot accurately describe the image information, so that the classification result will be unsatisfactory. However, the RmRMR visual word selection algorithm weights the words, and the words with the highest weights and greatest contributions to the classification are retained, while the redundancy and relevance between word and class are globally considered. Hence, the image classification accuracy will be improved. In addition, if the visual dictionary contains more than 1,400 entries, the classification precision of the two algorithms tends to decrease because the visual dictionary is too large and will classify similar image objects into two classes, thus increasing the calculating load and reducing the classification precision. The best effect will be achieved if the dictionary subset parameter of the proposed algorithm *d* is set to 800.

In this section, image patches are selected from a remote sensing image of the experimental area: 150 images of five classes are selected. The purpose is to train the proposed classification algorithm and evaluate its classification precision. The effects of different numbers of training samples on the classification accuracy are compared through experiment. To carry out the experiment, 40, 60, 80, 100, and 120 training samples are selected for each image class; other data are kept the same. The average classification accuracy of 20 times experiments is selected as the evaluation result. Furthermore, to validate the effectiveness of the proposed algorithm, the classic BoVW algorithm is selected for reference, and an image is classified using different numbers of training samples.

**Figure 4:**
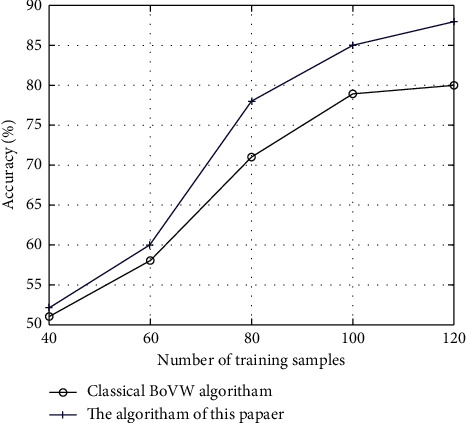
Results of the comparison experiment for different numbers of training samples.

The experimental results are as shown in Figure 4. With the increasing number of training samples, the classification precision for the two algorithms improves continuously. If the number of training samples is more than 40, the average classification accuracy of the proposed algorithm is higher than that of the classic BoVW algorithm and has a more distinctive tendency. The analysis indicates that if the number of training samples is small, the model learning is inadequate to describe the image information globally, leading to poor classification effect. On the other hand, more samples for training obtain higher classification accuracy. However, as the number of samples increases, the data redundancy is increasing and the classification precision tends to plateau. The proposed classification algorithm optimizes the process of feature extraction of a remote sensing image. Specifically, it extracts multifeature of the image and captures the spatial structure information from different objects. As a result, the algorithm shows a stronger ability in feature representations, and even with a small sample, its classification accuracy is better than that of the classic BoVW algorithm. The best performance will be achieved if the number of training samples is set to 800 in this experiment.

Section 3 introduces a weighting factor *α* into the proposed dictionary discriminant function to balance the redundancy between words and the relevance between words and classes. This section examines the effect of the weighting factor on classification performance. In the experiment, *α* = 0, 0.2, 0.4, 0.6, 0.8, and 1 are selected as the experimental hyperparameters, while other parameters are kept the same, and the classification accuracy for different values of is compared.

**Figure 5:**
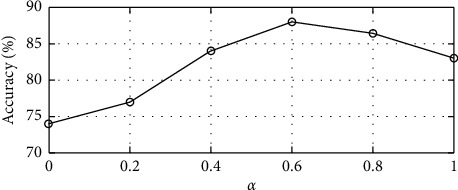
Results of the comparison experiment for different values of the weighting factor.

The experimental results are shown in Figure 5. The classification accuracy is the highest when *α* = 0.6 and the lowest when *α* = 0. The analysis indicates that the effect of the relevance between words and classes on the classification result is greater than that of the redundancy between words on the classification result for the high-resolution remote sensing image. In addition, the classification accuracy is the lowest when *α* = 0, namely, when the relevance between words and classes is disregarded. The algorithm selects visual words by calculating the mutual information when *α*^=1,^ namely, when the redundancy between words is disregarded. The algorithm balances the relevance and redundancy when *α* = 0.5, but the classification result is not the best. The best classification result is achieved when *α* =0.6 for the high-resolution remote sensing image. Therefore, the RmRMR algorithm will select more words with high relevance between words and classes to obtain the globally discriminative dictionary subset, and a few redundant words are allowed in the dictionary.

### 4.3. Qualitative Results

To verify the effectiveness and complexity of the proposed algorithm, this section carries out a classification experiment on the same high-resolution remote sensing image using different methods: pixel-based classification [15], classic BoVW classification [16], object-based classification [17], features-based classification [18], and the proposed classification.

The classification experiment is carried out with the above methods. For the proposed algorithm, the texture features are extracted with the grey-level cooccurrence matrix (GLCM), four features (entropy, homogeneity, nonsimilarity, and angular second moment) are selected, and the size of the neighboring window is 5×5 with a step length of 1. For the classic BoVW algorithm, the low-level features are extracted with SIFT and spectral features, the number of training samples is 120, the dictionary size is 800, and the RBF kernel function is selected in the SVM classifier. The object feature-based SVM algorithm also selects SIFT and spectral features, and the kernel function is RBF. Other experimental parameters are kept the same.

**Figure 6:**
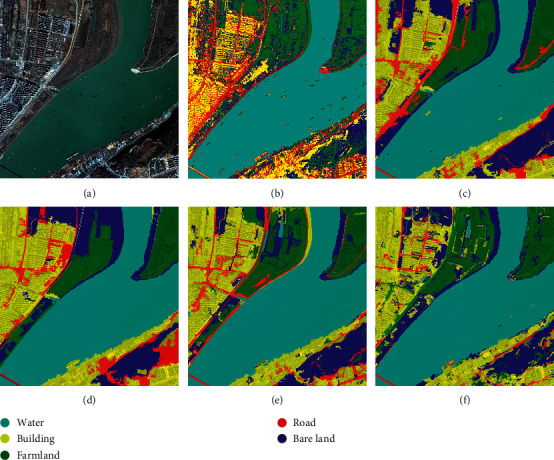
Comparison of the experimental results. (a) Original image, (b) pixel-based classification, (c) classic BoVW classification, (d) object-based classification, (e) features-based classification, and (f) the proposed classification.

According to the comparison results in Figure 6, the pixel-based maximum likelihood classification method is vulnerable to misclassifications and trivial salt as well as pepper noise, while the other four object-oriented classification methods yield more complete and clear results that are more consistent with the human visual experience, almost with no salt and pepper noise. The pixel-based classification algorithm (Figure 6(a)) maintains uniformity for the ground object with a larger area because it uses individual spectral features and performs classification in units of pixels; however, it cannot accurately and globally represent the information of a high-resolution remote sensing image. The object-oriented classification algorithms effectively overcome these deficiencies. The comparison between Figures 6(c) and 6(f) indicates that the result of the classic BoVW classification algorithm contains misclassifications and omits objects, because words cannot be mapped to the correct classes if the relevance between visual words and classes is inadequate or if the redundancy between words is high compared to the proposed algorithm. The comparison between Figures 6(e) and 6(f) indicates that the features-based classification and the proposed algorithm yield similar classification results. Features-based classification generates a set of visual words that are used to encode image features through the N-Gram model, fuses the perceived shape and spatial distribution features, and achieves satisfactory results. In contrast, the proposed algorithm yields classification results with clearer edges and details and can accurately distinguish ground objects because it uses the anisotropic diffusion filter at the image preprocessing stage. In addition, it selects KAZE as one of the low-level features at the feature extraction stage, highlights the contours of image edges, and effectively overcomes the problem of fuzzy boundaries and lost details.

### 4.4. Quantitative Results

This section introduces the precision evaluation criterion, calculates the Kappa coefficient and overall classification precision to compare the performance of the above five algorithms, and gives the confusion matrix of the proposed algorithm.

**Figure 7:**
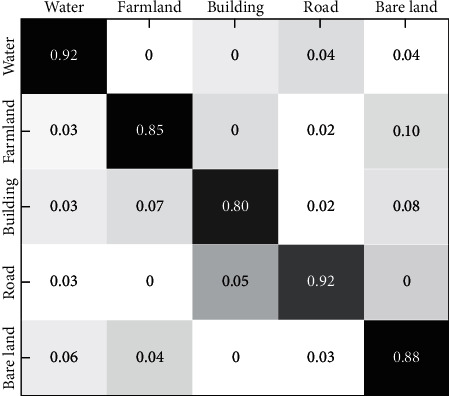
Confusion matrix of the proposed algorithm.

Figure 7 shows the confusion matrix of the proposed algorithm. It shows that “farmland” and “bare land” are easily confused by the proposed algorithm, because they contain many similar feature points. In addition, the image content that is contained in “building” is complex and variant, and the individual classification accuracy is only 80%; therefore, it is easily misclassified as “bare land”. In contrast, the ground object information contained in “road” and “water” is relatively simple, and the classification accuracy reaches 92%.

To further demonstrate the effectiveness and complexity of the proposed algorithm, the overall classification precision, Kappa coefficients, and total running times for the different algorithms are given in Table 1. According to the overall classification precision and Kappa coefficient, the classification effect of the proposed algorithm is the best, while the pixel-based maximum likelihood algorithm is the poorest. The proposed algorithm is an object-oriented classification method. In the stage of image feature extraction and representation, the BoVW algorithm is optimized to extract and fuse multiple features, and this has improved the classification precision. However, the computational load is increased as the total running time of the proposed algorithm is approximately 300s slower than that of the classic BoVW algorithm. On the other hand, its overall classification precision is 2.1% and 33.3% higher than those of the method features-based classification and the pixel-based maximum likelihood classification algorithm, respectively.

## 5. Conclusions

This paper proposes the BoVW-based high-resolution remote sensing image classification method. Using the RmRMR algorithm, it establishes a dictionary determinant function to filter the dictionary subset with minimum redundancy between words and maximum relevance between words and classes. Experimental results show that the proposed algorithm globally and accurately describes the image content and bridges the gap between high- and low-level features. Also, it improves the discriminative power of the dictionary and can reduce redundant information compared to the traditional classification algorithms. The final average classification accuracy on a remote sensing image is 88%. The proposed method provides a novel approach to high-resolution remote sensing image classification. Future work will apply the proposed algorithm to the aerial video scene classification task, and we will improve the algorithm by leveraging the advantages of Recurrent Neural Network (RNN) in dealing with dynamic timing problems.

## Figures and Tables

**Table 1:** Comparisons of the classification performance of different algorithms.

	Pixel-based classification	Classic BoVW classification	Object-based classification	Features-based classification	The proposed classification
Running total time (s)	57	460	291	553	767
Overall classification accuracy (%)	54.9	79.0	71.8	86.1	88.2
Kappa coefficient	0.47	0.74	0.68	0.81	0.84

## Data Availability

The US GeoEye-1 remote sensing image dataset used to support the findings of this study is available at https://www.satimagingcorp.com/gallery/geoeye-1/.
